# Complete plastid genome sequence of *Halimodendron halodendron* (Leguminosae)

**DOI:** 10.1080/23802359.2021.1920501

**Published:** 2021-07-20

**Authors:** Wen-Jie Yan, Tian-Ge Yang, Er-Dai Qin, Wen-Rui Qu, Zhi-Hua Wu, Pei-Pei Jiao, Hong Liu

**Affiliations:** a College of Biochemical Engineering, Beijing Union University, Beijing, China; b Hubei Provincial Key Laboratory for Protection and Application of Special Plant Germplasm in Wuling Area of China, College of Life Sciences, South-Central University for Nationalities, Wuhan, China; c College of Life Science, Tarim University, Alar, China; d Xinjiang Production & Construction Corps Key Laboratory of Protection and Utilization of Biological Resources in Tarim Basin, Tarim University, Alar, China; e College of Life Science and Technology of Huazhong Agricultural University, Wuhan, China

**Keywords:** *Halimodendron halodendron*, chloroplast genome, phylogeny

## Abstract

*Halimodendron halodendron* (Pall.) Voss. is a deciduous shrub belonging to the genus *Halimodendron*, Leguminosae, and is mainly distributed in dry areas. This species can be used for saline-alkali soil improvement and sand fixation. The complete plastid genome of *H. halodendron* first reported here is 129,342 bp in length, and contains 110 genes, including 76 protein coding genes, 30 tRNA genes, and 4 rRNA genes. A total of 105 simple sequence repeats (SSRs) were identified in the chloroplast genome. This information will be useful for study on the evolution and genetic diversity of *Halimodendron halodendron* in the future.

*Halimodendron halodendron* (Pall.) Voss. is a deciduous shrub belonging to Leguminosae. It mainly grows in Xinjiang, Gansu (sandy soil of Hexi Corridor), northwestern Inner Mongolia in China, and the former Soviet Union and Mongolia also have distribution. It is usually found in desert salinized sandy soils, saline soils along riverbank, and under *Populus euphratica* forests. *Halimodendron halodendron* has significant effects on soil nitrogen fixation under arid or semi-arid conditions (Matinkhah et al. 2020). Besides, the phenols extracted from *H. halodendron* also show antibacterial and antioxidant activities (Wang et al. 2012). Therefore, it is both an ecologically and economically important plant. In this study, to obtain the new insight into the phylogeny of *H. halodendron*, we assembled and annotated the plastid genome from sequenced data.

The materials of *H. halodendron* in this study were collected from Wushi County, Aksu prefecture, Xinjiang province of China (79°24.949′E, 41°12.346′N, 1322 m above sea level). The voucher specimen (TD-00549, *Halimodendron halodendron* (Pall.) Voss.) was stored in the herbarium of Tarim University, and the data related to the specimens are included in the database of wild plant germplasm resources of the Tarim basin (http://res.taru.edu.cn/, internal website, not yet open to the public; data available from the corresponding author Peipei Jiao upon reasonable request, jiaopeipei2000@126.com). The complete genomic DNA was extracted using CTAB method (Doyle et al. 1987) and sequenced using the Illumina NovaSeq 6000 platform at Majorbio Company (Shanghai, China). We removed low-quality sequences from the raw data (SRR13270803). The trimmed reads were assembled using GetOrganelle v1.7.3 (Jin et al. 2020). Then, the plastid gene structures were annotated using CPGAVAS2 (Shi et al. 2019) and PGA (Qu et al. 2019). The complete plastid genome was 129,342 bp (MW349012) and lost an IR region, the average GC content was 34.4%. Generally, chloroplast genomes are characterized by a quadripartite structure, with two copies of an inverted repeat (IR) separating the large (LSC) and small (SSC) single copy regions. Losing one copy of the IR in the plastid genome is a common phenomenon in some tribes among legumes, such as Carmichaelieae, Cicereae, Hedysareae, Trifolieae, Fabeae, Galegeae, and three genera of Millettieae (Palmer and Thompson 1982; Lavin et al. 1990; Liston 1995; Jansen et al. 2008). This may be a special feature of legumes in the evolutionary process. The complete plastid genomes encoded 110 functional genes, including 76 protein-coding genes, 30 tRNA genes, and 4 rRNA genes. A total of 105 SSR markers ranging from mononucleotide to hexa-nucleotide repeat motif were identified in *H. halodendron* plastid genome.

To explore the phylogenetic relationship of *H. halodendron* within Leguminosae, additional 23 species from Leguminosae were studied. With the *Polygala tenuifolia* and *Polygala fallax* as the outgroups, the phylogenetic trees were built from the whole protein-coding gene matrix by maximum-likelihood (ML) and Bayesian inference (BI) (Figure 1). The ML tree was generated using IQ-TREE v2.1.2 (Nguyen et al. 2015) based on the best model of TVM + F+G4 and 1000 bootstrap replicates, and BI analysis was performed in MrBayes v3.2.7 (Ronquist et al. 2012). *Halimodendron halodendron* is the only species under the genus of *Halimodendron* currently, and the phylogenetic trees indicate that *H. halodendron* was closer to the species of *Caragana kozlowii.* The information will provide the basis for the study of *H. halodendron* in the future.

**Figure 1.**
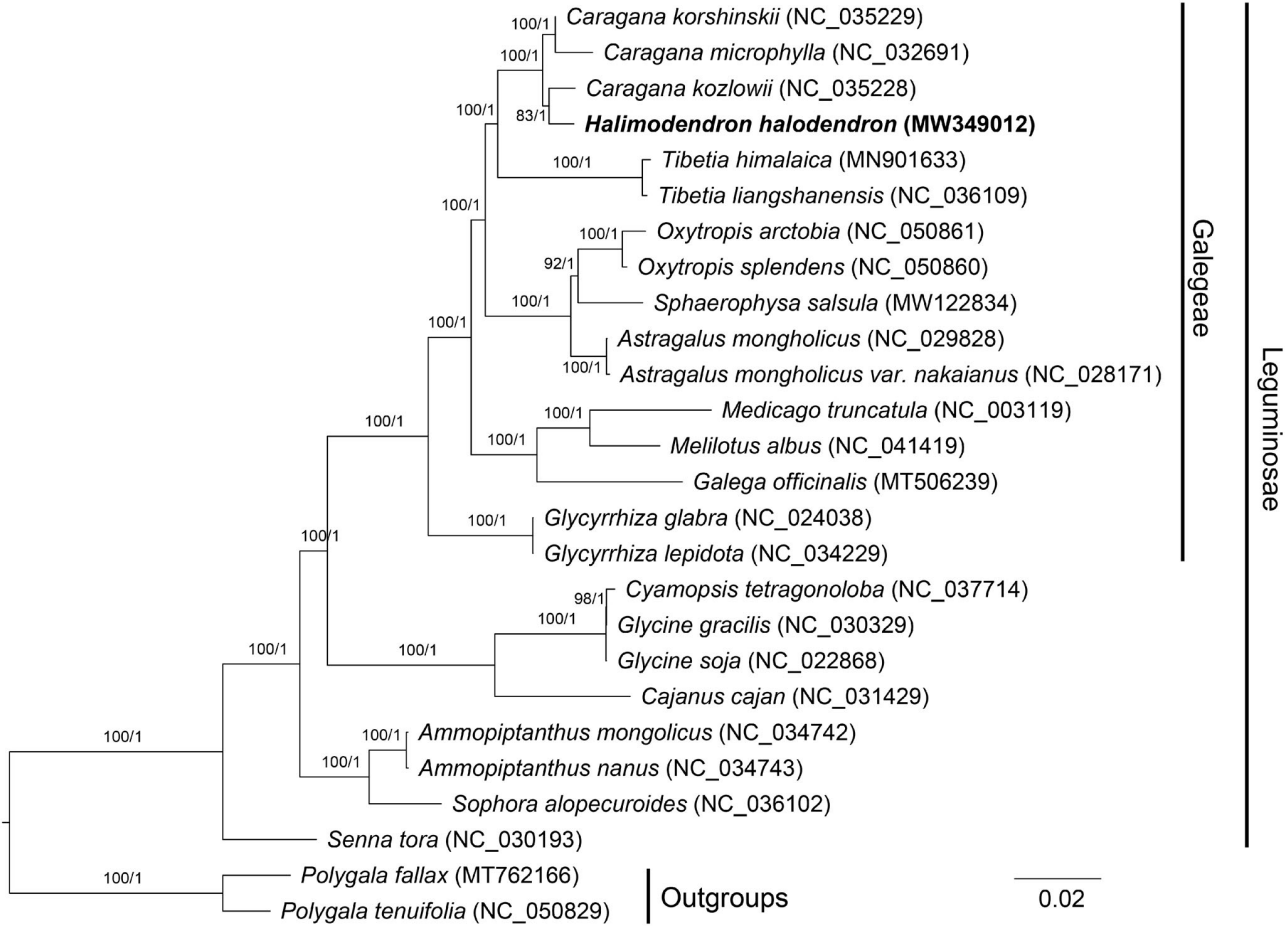
Phylogenetic tree reconstructed by maximum-likelihood (ML) and Bayesian inference (BI) analysis based on the 79 plastid protein-coding genes of 26 species. Values above branches are maximum likelihood bootstrap percentages (BS)/Bayesian posterior probabilities (PP).

## Data Availability

The genome sequence data that support the findings of this study are openly available in GenBank of NCBI at (https://www.ncbi.nlm.nih.gov) (https://www.ncbi.nlm.nih.gov/) under the accession no. MW349012. The associated “BioProject,” “SRA,” and “Bio-Sample” numbers are PRJNA686216, SRR13270803, and SAMN17109175, respectively.

## References

[CIT0012] Doyle JJ, Doyle JL. 1987. A Rapid DNA isolation procedure from small quantities of fresh leaf tissues. Phytochem Bull. 19:11–15.

[CIT0001] Jansen RK, Wojciechowski MF, Sanniyasi E, Lee SB, Daniell H. 2008. Complete plastid genome sequence of the chickpea (*Cicer arietinum*) and the phylogenetic distribution of *rps12* and *clpP* intron losses among legumes (Leguminosae). Mol Phylogenet Evol. 48(3):1204–1217. 18638561 10.1016/j.ympev.2008.06.013 PMC2586962

[CIT0002] Jin J-J, Yu W-B, Yang J-B, Song Y, dePamphilis CW, Yi T-S, Li D-Z. 2020. GetOrganelle: a fast and versatile toolkit for accurate de novo assembly of organelle genomes. Genome Biol. 21(1):241. 32912315 10.1186/s13059-020-02154-5 PMC7488116

[CIT0003] Lavin M, Doyle JJ, Palmer JD. 1990. Evolutionary significance of the loss of the chloroplast–DNA inverted repeat in the Leguminosae subfamily Papilionoideae. Evolution. 44(2):390–402. 28564377 10.1111/j.1558-5646.1990.tb05207.x

[CIT0004] Liston A. 1995. Use of the polymerase chain reaction to survey for the loss of the inverted repeat in the legume chloroplast genome. In: Crisp M, Doyle JJ, editors. Advances in legume systematics 7: phylogeny. Kew: Royal Botanic Gardens; p. 31–40.

[CIT0005] Matinkhah SH, Yazdanshenas H, Sheikhizadeh M. 2020. Nitrogen-fixing potential of *Halimodendron halodendron* (Pall.) Voss in arid and semi-arid areas of Iran. SN Appl Sci. 2(11):10.

[CIT0006] Nguyen LT, Schmidt HA, von Haeseler A, Quang MB. 2015. IQ-TREE: a fast and effective stochastic algorithm for estimating maximum-likelihood phylogenies. Mol Biol Evol. 32(1):268–274. 25371430 10.1093/molbev/msu300 PMC4271533

[CIT0007] Palmer JD, Thompson WF. 1982. Chloroplast DNA rearrangements are more frequent when a large inverted repeat sequence is lost. Cell. 29(2):537–550. 6288261 10.1016/0092-8674(82)90170-2

[CIT0008] Qu XJ, Moore MJ, Li DZ, Yi TS. 2019. PGA: a software package for rapid, accurate, and flexible batch annotation of plastomes. Plant Methods. 15(1):50. 31139240 10.1186/s13007-019-0435-7 PMC6528300

[CIT0009] Ronquist F, Teslenko M, van der Mark P, Ayres DL, Darling A, Höhna S, Larget B, Liu L, Suchard MA, Huelsenbeck JP. 2012. MrBayes 3.2: efficient Bayesian phylogenetic inference and model choice across a large model space. System Biol. 61(3):539–542. 22357727 10.1093/sysbio/sys029 PMC3329765

[CIT0010] Shi L, Chen H, Jiang M, Wang L, Wu X, Huang L, Liu C. 2019. CPGAVAS2, an integrated plastome sequence annotator and analyzer. Nucleic Acids Res. 47(W1):W65–W73. 31066451 10.1093/nar/gkz345 PMC6602467

[CIT0011] Wang JH, Lou JF, Luo C, Zhou LG, Wang MG, Wang L. 2012. Phenolic compounds from *Halimodendron halodendron* (Pall.) Voss and their antimicrobial and antioxidant activities. IJMS. 13(9):11349–11364. 23109858 10.3390/ijms130911349 PMC3472750

